# 

**Published:** 1890-04

**Authors:** 


### CONTENTS.

COMMUNICATIONS.

Imagination .... 161
Inventions and New Things .... 165
The Wealth of Dentists .... 170
Non-Metallic Plastic Materials for Filling Teeth .... 175
President's Address .... 180
Professional Ethics and Honor .... 183
Evolution in Dentistry .... 191
Magnesii Sulphas .... 197

SELECTIONS.

A National Department of Public Health .... 199
The Regulation of Sleep .... 200
Facial Neuralgia and Allied Neurosis .... 201
Death in Soothing Syrup .... 202
Bromide of Lithia .... 202
A Paste that Will Adhere to Anything .... 203

CORRESPONDENCE.

Letter from Geo. A. Mills, New York .... 204

NOTICES.

American Medical Association .... 205
Programme of the Dental Section .... 207
International Medical Congress of 1890 .... 207
Report of the Committe on Foreign Transportation .... 208
Tenth International Medical Congress—Preliminary Programme of the Dental Section .... 209

EDITORIAL.

Experiments with the Electro-Deposit Plate .... 210
The Chase Metallic Combination Plate .... 211
What About It? .... 211
Transactions of the American Dental Association .... 212
Obituary .... 212

